# Supplementary material for: Peptidyl Transferase Center and the Emergence of the Translation System
Source: Life (Basel). 2017 Apr 25;7(2):21. doi: 10.3390/life7020021 (PMC5492143; doi:10.3390/life7020021)
Supplement: Supplementary file 1 [file life-07-00021-s001.pdf]

Supplementary Martials: Peptidyl Transferase Center and the Emergence of the Translation System

Savio Torres de Farias, Thais Gaudêncio Rêgo and Marco V. José

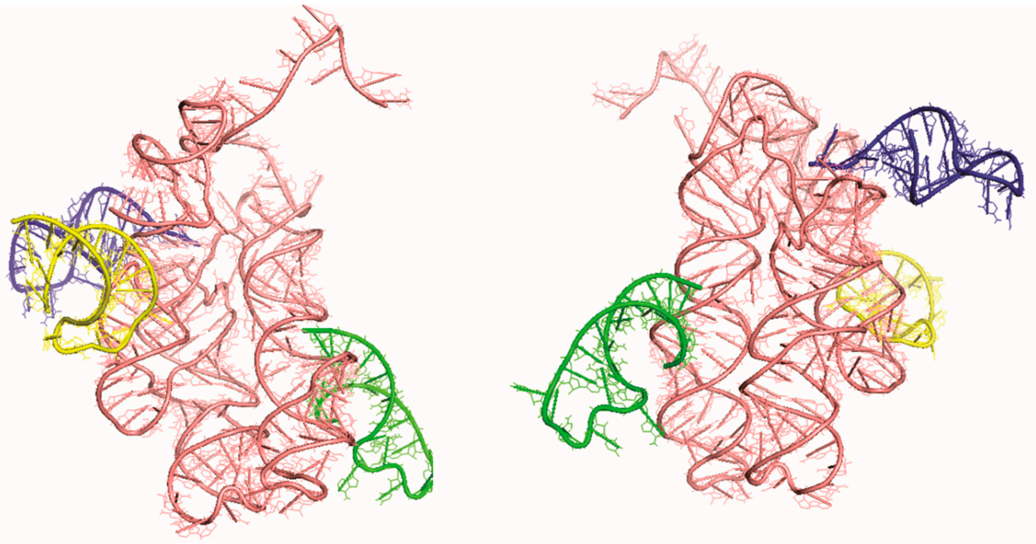

Figure 1. Two different perspectives of the superimposition of the docking experiments between the *T. thermophilus* Peptidyl Transferase Center (PTC) (salmon) and $tRNA^{Gly}$ (blue), $tRNA^{Arg}$ (green), and $tRNA^{Asp}$ (yellow) anticodon loop, with rotation of 180 degrees between them.
